# Does anticipation of penalty kicks in soccer transfer across similar and dissimilar sports?

**DOI:** 10.1007/s10339-021-01073-y

**Published:** 2022-03-30

**Authors:** Matthew Andrew, Joe Causer

**Affiliations:** Faculty of Science, Research Institute for Sport and Exercise Sciences, Liverpool John Moores University, Liverpool, UK

**Keywords:** Specificity, Skill acquisition, Perceptual-cognitive skill, Expertise

## Abstract

The aim of the present study was to examine whether anticipation skill associated with penalty-kick scenarios is sport-specific, or whether it transfers between sports that have similar elements. A shortened participation history questionnaire was used to identify 97 soccer players, 47 invasion sport players (e.g., rugby), and 72 other sport players (e.g., swimming), as well as skill level (hours of engagement/competition level). These participants completed a video-based temporal occlusion anticipation test that required them to select the destination of the ball across a series of soccer penalty scenarios. Results indicated that the skilled soccer players were more accurate than the skilled and less-skilled invasion sport players and skilled and less-skilled other sport players. Skilled soccer players were also more accurate than the less-skilled soccer players, with less-skilled soccer players exhibiting similar accuracy to both the skilled and less-skilled invasion sport and other sport players indicating that processes associated with anticipation of penalty kicks may be specific to their sport.

## Introduction

Anticipation is fundamental to expert performance in highly dynamic, time-pressured sports such as soccer (Williams and Jackson 2019). Anticipation refers to an athlete’s ability to predict what events and/or actions are likely to unfold prior to an event occurring (Williams and Jackson 2019). Over the years, researchers have examined the processes and mechanisms that facilitate successful anticipation in soccer (Causer et al. 2017) yet investigations as to whether these processes resulting from one sport can transfer to another are currently limited (Müller and Rosalie 2019). The study of transfer could provide useful understanding into expert performance and whether domain-specific knowledge acquired from one sport can transfer to similar or dissimilar sports (Smeeton et al. 2004). Thorndike (1914) first put forward the idea of transfer through his notion of identical elements. It was posited that the level of successful transfer is dependent on the level or amount of similar (i.e., identical) elements (e.g., motor; perceptual; conceptual) between the two performance or domains. Soccer and other invasion sports (e.g., hockey) contain similar perceptual (e.g., tracking ball flight) and tactical elements (e.g., patterns of play) suggesting a bi-directional transfer could occur between the two sports (Smeeton et al. 2004). On the contrary, sports that do not transfer to another domain (i.e., invasion vs. net sports) suggest specificity of learning (Proteau 1992).

Evidence of positive transfer of perceptual-cognitive skills across sports have previously been observed (Rienhoff et al. 2013; Moore and Müller 2014; Rosalie and Müller 2014; Roca and Williams 2017). For instance, in our previous work, we compared anticipation ability of skilled and less-skilled players from soccer, invasion sports, and other sports during a video-based temporal occlusion soccer decision-making test (4 vs. 4 soccer scenarios; Causer and Ford 2014). Skilled soccer players were more accurate compared to less-skilled soccer players. Moreover, no differences were observed between the soccer players and other invasion sports players that share similar elements, who both outperformed the other sports group that do not share these elements, providing support for positive transfer of anticipation. There are, however, some contexts where the transfer of perceptual-cognitive skills is specific to their sport. For example, skilled and less-skilled soccer players, other invasion sport players, and other sport players completed a video-based temporal occlusion test designed to measure decision-making in 11 versus 11 defensive soccer scenarios. It was observed that skilled soccer players were more accurate than the invasion sport players and other sport players. Skilled soccer players were also more accurate than the less-skilled soccer players, that exhibited similar accuracy to both the skilled and less-skilled invasion sport and other sport players (Andrew et al. 2021). These findings indicate that in some scenarios in soccer, transfer of perceptual-cognitive skills to similar sports may be restricted, and thus expert performance may only be achieved through sport-specific practice.

Previous examinations of the transfer of perceptual-cognitive skills, particularly from soccer to similar and dissimilar sports have employed sequences from ‘open-play’ scenarios where the options are either to pass or tackle, etc. (Causer and Ford 2014; Roca and Williams 2017; Andrew et al. 2021). However, during soccer, there are many scenarios where the ball is ‘out-of-play’ and must be ‘restarted’, such as a free-kick or penalty-kick, which account for 30–40% of all actions in a soccer match (Yiannakos and Armatas 2006). The temporal constraints of these scenarios differ from open-play, with goalkeepers typically having under 1.5 s to anticipate the direction and flight of the ball (Dicks et al. 2010; Causer et al. 2017). Research examining perceptual-cognitive skills during a penalty-kick scenario in soccer reported skilled goalkeepers’ anticipation responses were more accurate compared to their less-skilled counterparts (Causer et al. 2017), yet the examination of whether anticipation skills can transfer across similar or dissimilar sports remain limited.

The aim of the present study was to examine whether anticipation associated with penalty kick scenarios transfer between sports that have similar elements, and those that do not. Skilled and less-skilled participants from three sporting groups (soccer; invasion sports; other sports) completed a soccer anticipation test. Invasion sports were defined as those that require teams to score points in goals and lines positioned at the end of the pitch behind the opposition team (e.g., rugby, basketball), while other sports included classifications such as athletics, net/wall games (e.g., badminton), striking/fielding games (e.g., cricket) and target sports (e.g., golf) (Launder 2001). It was expected that if processes associated with anticipation skill during a penalty-kick are transferable, skilled soccer players would be better at anticipation compared to players of dissimilar sports, yet not compared to those from similar sports. If, however, these processes are sport-specific, skilled soccer players would be better at anticipation compared to players from both similar and dissimilar sports.

## Method

### Volunteers

Participants were 216 undergraduate sport science students recruited from the student body within the School of Sport and Exercise Science at the host university. All procedures were conducted in accordance with the ethical guidelines of the host institution. 97 outfield soccer players, 47 outfield other invasion sport players (rugby = 16; netball = 14; basketball = 8; hockey = 4; Gaelic = 3; lacrosse = 2) and 72 other sport players (athletics/gymnastics = 36; net/wall games = 11; striking/fielding = 7; water sports = 7; combat sports = 7; horse = 2; target sports = 2) were identified based on their participation data collected via a shortened version of the Participation History Questionnaire (Ford et al. 2010). No other invasion sport or other sport players had engaged in soccer, or play in a goalkeeper position in other sports that require one (e.g., hockey). We used outfield players to attain a larger sample size, as previous studies examining anticipation during penalty-kicks used relatively small sample sizes (*n* = 14, Savelsbergh et al. 2002; *n* = 8, Dicks et al. 2010). In each of these classifications, participants were further divided into 98 skilled (e.g., national) players and 118 less-skilled (e.g., regional) players based upon a combination of current and/or highest level of performance and hours of engagement in their primary sport (Swann et al. 2015). Many of the skilled players were representing the host universities in their respective sport and engaged in total of 4200.7 (*sd* = 1983.9) hours in their primary sport, with 1187.6 (*sd* = 595.0) hours for the less-skilled players (Table 1).

**Table 1:** Mean (*sd*) characteristics of each group and skill level

Group	Skill	Sport/Class	*n*	No. Oth sports	Total hrs in prim sport
Soccer	Skilled		38	5.6 (3.4)	4946.9 (2165.8)
	Less-skilled		59	4.3 (2.3)	1719.6 (697.2)
Invasion	Skilled		23	5.8 (2.2)	2672.0 (867.9)
		Rugby	10	5.3 (1.5)	2761.7 (703.5)
		Netball	7	6.4 (3.0)	2882.0 (1298.2)
		Basketball	4	6.8 (1.5)	2536.3 (220.3)
		Hockey	1	6.0	1454.9
		Gaelic	1	2.0	3117.6
	Less-skilled		24	4.6 (2.9)	849.8 (410.3)
		Rugby	6	3.8 (1.9)	785.2 (408.1)
		Netball	7	4.7 (3.8)	1115.9 (208.9)
		Basketball	4	3.5 (1.0)	889.8 (367.5)
		Hockey	3	8.7 (0.6)	1021.9 (494.7)
		Gaelic	2	1.5 (0.7)	311.8 (147.0)
		Lacrosse	2	6.0 (1.4)	311.8 (293.9)
Other sports	Skilled		37	5.2 (2.2)	4983.2 (2918.0)
		Athletics/gym	16	5.3 (1.9)	4817.7 (3770.6)
		Strike/field	7	4.6 (3.1)	4607.1 (2256.7)
		Net games	5	5.6 (1.8)	5022.8 (1746.2)
		Watersports	4	3.8 (2.2)	4689.4 (1702.5)
		Aiming	2	8.0 (2.8)	4156.8 (0.0)
		Combat	2	5.0 (0.0)	5975.4 (1249.2)
		Horse	1	5.0	10,911.6
	Less-skilled		35	5.1 (2.1)	993.3 (677.6)
		Athletics/gym	20	5.1 (1.8)	863.6 (595.0)
		Net games	6	6.5 (2.6)	1225.4 (726.8)
		Watersports	3	4.7 (2.1)	1388.5 (952.6)
		Combat	5	4.0 (2.3)	696.3 (389.4)
		Horse	1	3.0	2494.1

### Procedure

**Fig. 1:**
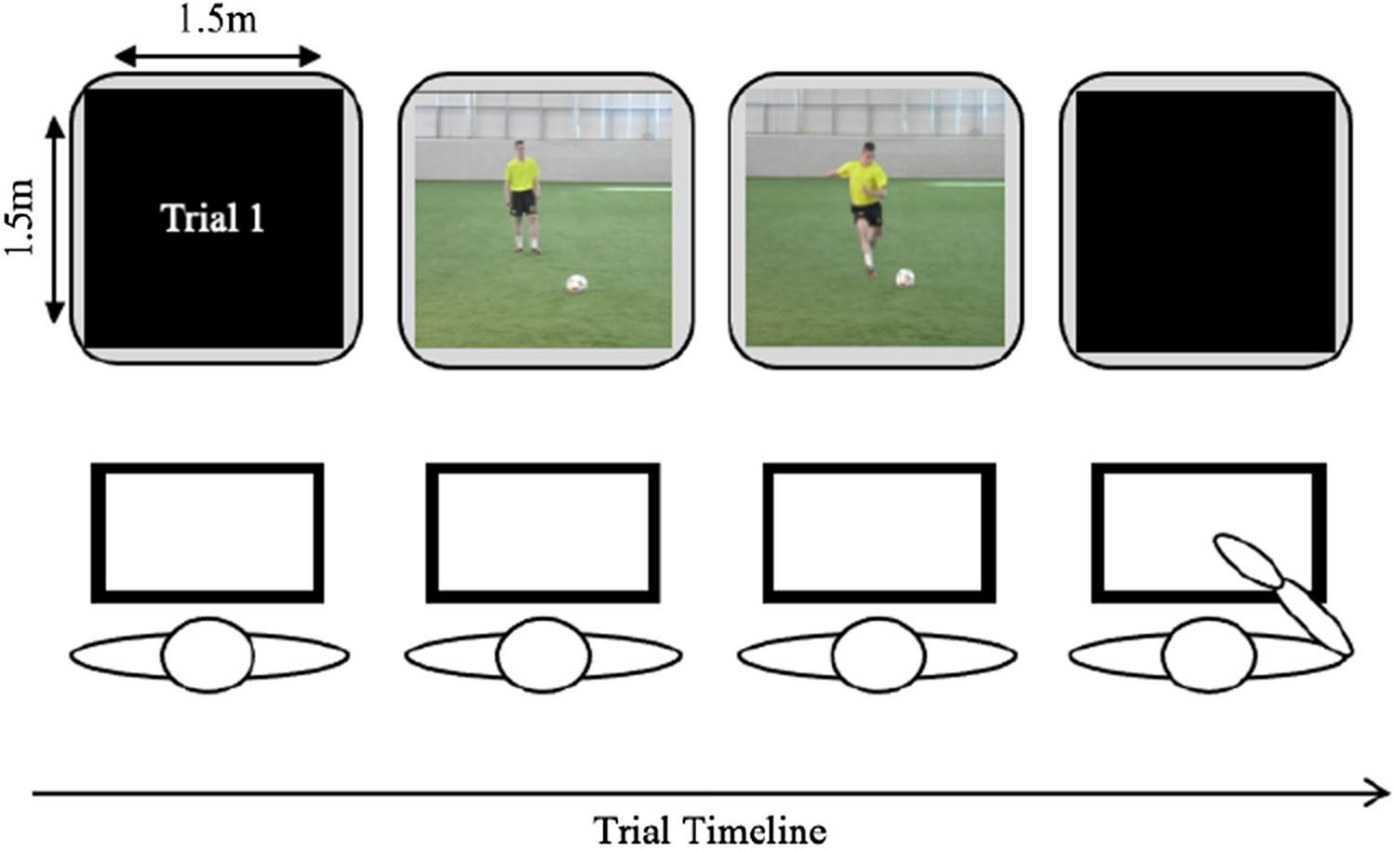
A schematic representation of the experimental set-up for the video-based anticipation test of the penalty-kick scenario

**Fig. 2:**
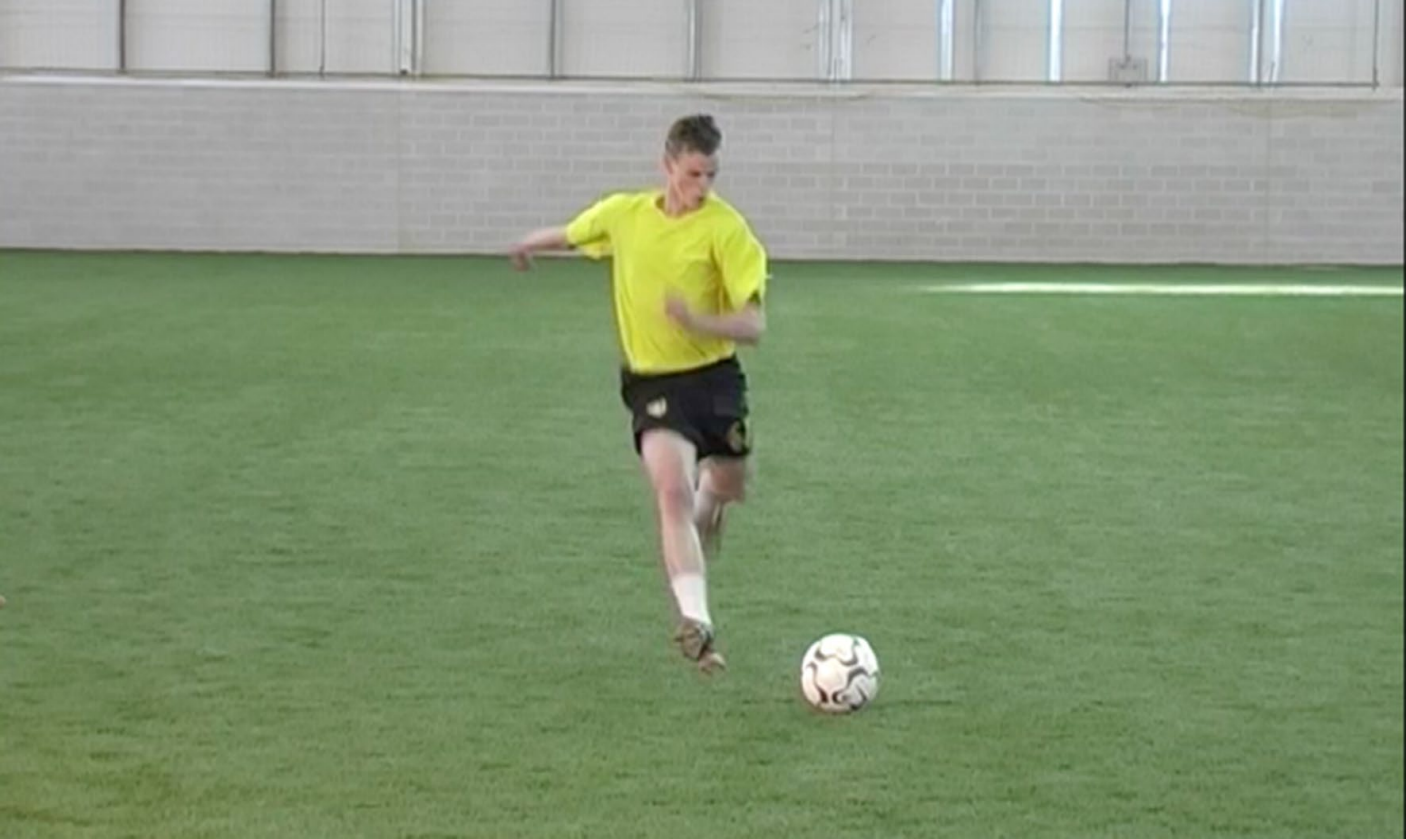
Example of a frame from the video-based anticipation test of the penalty-kick scenario, which demonstrates the perspective of the participant

All participants completed a video-based temporal occlusion anticipation test in which they were required to predict the outcome of the penalty kick. The task was akin to Causer et al. (2017) examining anticipation skill in soccer penalty-kicks. The participants viewed soccer footage that was life-sized on a large video screen (1.5 m × 1.5 m, 0.5 m from floor to bottom of screen; Fig. 1) and from the first person perspective of a goalkeeper who was facing a penalty-kick (Fig. 2). Each video clip started with a countdown (starting at 3) to allow the participants time to prepare, followed by an opponent running up to take a single shot on goal. Each video clip lasted between 2–3 s and ended when an opaque screen occluded the video at ball contact. The opaque screen remained for approximately 4 s (consistent across all trials). During this time, participants were required to confirm the destination of the ball and thus the direction of travel to save/defend the penalty based on four options (top left, bottom left, top right, bottom right; Fig. 1). Participants were instructed to select their response as quickly as they could, to closely replicate the requirements of the task, yet the time taken to select their response was not recorded. The destination of the ball always contained one of these options and all shots were ‘on target’ whereby they would hit the net of the goal. There was a 25% chance that the participants would ‘guess’ the correct response. Participants completed 5 familiarisation trials (no feedback was provided to replicate the task) and 20 experimental trials. There was no repetition of trials within the experiment. All sessions were completed in approximately 15 min. The order of the presentation clips remained constant across all participants.

### Data analysis

Each participant was awarded a point for each correct answer. A total score was calculated for each participant and expressed as a percentage (%) for response accuracy. A two-way, between groups ANOVA with Bonferroni correction to adjust for familywise error, was conducted on the data to analyze response accuracy score with sport classification (soccer; invasion sports; other sports) and expertise (skilled; less-skilled) as the between-group factors. Any violations to sphericity were corrected using Huynh–Feldt procedures when the Greenhouse–Geisser value was greater than 0.75. Effect sizes were reflected as Partial eta squared ($\eta_{p}^{2}$) and Cohens *d* as appropriate. Significant main and/or interactions effects involving more than two means were analyzed using Tukey posthoc procedure. Thresholds for statistical significance were set at *p* < 0.05.

## Results

**Fig. 3:**
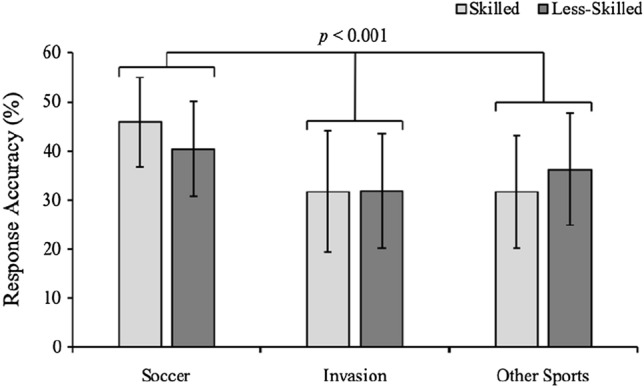
Mean response accuracy (%) in the soccer anticipation task for the skilled (light-grey bars) and less-skilled (dark-grey bars) soccer players, invasion sport players, and other sport players (error bars represent standard deviation)

Though there was no significant main effect of expertise [F(1, 210) = 0.023, *p* = 0.879, $\eta_{p}^{2}$ = 0.001], the ANOVA revealed a significant group x expertise interaction [F(2, 210) = 4.62, *p* = 0.01, $\eta_{p}^{2}$ = 0.14] indicating a significant difference in response accuracy between the groups dependent on expertise level. As observed in Fig. 3, skilled soccer players (*m* = 45.9%, *sd* = 9.1%) were significantly more accurate compared to skilled invasion sport players (*m* = 31.7%, *sd* = 12.3%, *p* < 0.01, *d* = 1.31) and skilled other sport players (*m* = 31.6%, *sd* = 11.5%, *p* < 0.01, *d* = 1.38). Furthermore, less-skilled soccer players (*m* = 40.4%, *sd* = 9.6%) were significantly more accurate compared to less-skilled invasion sport players (*m* = 31.9%, *sd* = 11.6%, *p* = 0.01, *d* = 0.80). Though close, there was no significant difference between less-skilled soccer players and less-skilled other sport players (*m* = 33.0%, *sd* = 9.9%, *p* = 0.07, *d* = 0.39). There was also a main effect of sport type [F(2, 210) = 23.587, *p* < 0.01, $\eta_{p}^{2}$ = 0.183]. Soccer players (*m* = 43%, *sd* = 9.3%) were significantly more accurate compared to the invasion (*m* = 31.8%, *sd* = 11.9%, *p* < 0.01, *d* = 0.99) and other sports players (*m* = 32.3%, *sd* = 10.7%, *p* < 0.01, *d* = 0.81), but there was no significant difference between the invasion and other sport players (*p* = 0.59, *d* = 0.18).

## Discussion

The aim of the present study was to examine whether anticipation skill associated with a penalty kick in soccer transfers between sports that share similar elements, or whether they are specific to their sport. As per previous literature and the identical elements theory (Thorndike 1914; Causer and Ford 2014), we hypothesized that skilled soccer players would be more accurate at decision-making than skilled other sport players that do not share similar elements but would not be when compared to skilled invasion sports players that do. If, however, these processes are specific to their sport (Müller et al. 2015; Andrew et al. 2021), then we hypothesized that skilled soccer players would be better at anticipation compared to skilled invasion and other sports players. In line with the latter hypothesis, findings indicated that there was no transfer of anticipation skills between similar or dissimilar sports, as the skilled soccer players were more accurate compared to the skilled invasion sport players and other sport players (Fig. 3), with differences of 14%. The response accuracy data indicates that underlying processes that underpin successful anticipation skill in a soccer penalty-kick scenario are likely to specific to their sport.

These findings are dissimilar to our previous work showing positive transfer of decision-making during ‘in-play’ scenarios (Causer and Ford 2014). This positive transfer was suggested to be associated with acquired visual search behaviors that underpin successful decision-making, which transferred across sports with similar elements. During these ‘in-play’ scenarios participants observed video clips between 4–5 s during which they had time to watch the actions unfold and thus make an appropriate response (Causer and Ford 2014; Roca and Williams 2017). Though eye movements were not captured during these studies it is likely that visual search strategies which involved fixations towards the opponent, teammates, space, and the player in possession of the ball (Roca et al. 2018, 2020). In comparison, consistent with a baseball pitch (Müller et al. 2015), anticipation of penalty kick are temporally constrained, with the goalkeeper typically having only 1.3–1.5 s during the run-up to anticipate the direction of the ball (Dicks et al. 2010). Therefore, to combat the temporal constraints, experts have developed sport-specific postural cues to predict oncoming ball trajectory which include fixations towards the kicking-leg, non-kicking-leg as well as the ball leading to successful anticipation (Dicks et al. 2010). Further support for sport-specific visual search strategies during temporally constrained tasks comes from ice-hockey (Panchuk and Vickers 2006), where the authors reported that successful anticipation ability was dependent on quiet eye (final fixation before movement onset) location(s) during shot preparation which was 1 s towards the stick/puck (70%) or the space in front of the puck (26%), yet very rarely on the body of the shooter (2%).

Soccer and other invasion sports are often considered ‘near’ transfer (Rosalie and Müller 2012) as they both require athletes to read patterns of play to anticipate whether to pass, dribble or shoot (Smeeton et al. 2004; Causer and Ford 2014; Roca and Williams 2017). The sequence of events of a soccer kick involves an attacking player approaching a stationary ball and takes a single shot (with their lower limbs) on a goal tended by a goalkeeper. In comparison, many other invasion sports involve penalty scenarios that do not contain a tended goal (e.g., rugby), and are upper limb dominant (e.g., basketball free throw), or do not feature kicking the ball (e.g., hockey penalty stroke/flick). The only other invasion sport that has a penalty scenario that is ecologically comparable is Gaelic football, yet they only represented 5% (*n* = 5) of the other invasion sport players. It could therefore be suggested that in this scenario that they are instead considered ‘far’ transfer, similar to the other sports players. Consistent with the findings of the present study, previous studies examining ‘far’, or dissimilar sports have consistently shown a lack of transfer of perceptual-cognitive skills (Smeeton et al. 2004; Abernethy et al. 2005; Moore and Müller 2014; Causer and Ford 2014; Müller et al. 2015; Roca and Williams 2017; Andrew et al. 2021).

Our findings suggest that experts develop and refine domain-specific memory structures the allow rapid and reliable retrieval of information from long-term memory (Ericsson and Kintsch 1995) that are sport-specific (i.e., penalty-kick), but sometimes with minimal adaptation that can be transferred to sports with similar elements. Although the mechanisms that facilitate successful anticipation skills are well known (Causer et al. 2017), future research should look to examine how these mechanisms contribute towards the specificity or transfer of perceptual-cognitive skills, as well as potential methods to expedite learning and transfer. These data also have practical implications for those responsible for the identification and development of talent (e.g., scouts). For instance, the annual turnover of academy soccer players in Germany is 25% (Güllich 2014). The released players may have the opportunity to recruited into other sports that share similar elements (i.e., talent transfer; Collins et al. 2014). It can be recommended that athletes should sample a variety of sports during their development to acquire general perceptual-cognitive skills (Causer and Ford 2014; Ford and Williams 2017), however in some scenarios such as a penalty-kick may require sport-specific practice to become expert performers (Güllich et al. 2020).

To summarise, our findings from the current study signify that anticipation skills during penalty-kick scenario are sport-specific, as skilled, and less-skilled soccer players response accuracy during an anticipation test was significantly higher than skilled and less-skilled other invasion sport players that share some similar elements (e.g., read patterns of play to anticipate whether to pass, dribble or shoot), and skilled and less-skilled other sport players that do not. A limitation to the present study is that our participants from the soccer groups were all outfield players, and thus characteristically do not face penalty-kicks. Though our findings do support some positive within-sport transfer (i.e., playing-position; Williams et al. 2008; Bruce et al. 2012), it could be anticipated that skilled goalkeeper’s response accuracy would be significantly higher. Indeed, using the same video clips as the current study, skilled goalkeepers exhibited ~ 90% response accuracy (Causer et al. 2017). We recommend that future work attempt to examine transfer of anticipation skill across similar sports that also contain penalty scenarios such as hockey, using goalkeepers only to better understand the underlying mechanisms that either account for the transferable (Causer and Ford 2014) or sport-specific (Andrew et al. 2021) perceptual-cognitive skills. Furthermore, though the present study contained other invasion sport players, most of these athletes’ represented sports that had penalty scenarios that were dissimilar to soccer (e.g., basketball, rugby, netball). To further examine transfer anticipation skills during penalty scenarios across similar sports (i.e., ‘near’ transfer; Rosalie and Müller 2012), we recommend that future work include sports that have penalty scenarios that share similar elements such as being tended by a goal (e.g., hockey, lacrosse Gaelic football).

## Data Availability

The data that support the findings of this study are available on request from the corresponding author.
